# Cancer-associated fibroblasts as accomplices to confer therapeutic resistance in cancer

**DOI:** 10.20517/cdr.2022.67

**Published:** 2022-09-07

**Authors:** Wenyu Wang, Bing Cheng, Qiang Yu

**Affiliations:** ^1^Guangdong Provincial Key laboratory of Colorectal and Pelvic Floor Disease, The Sixth Affiliated Hospital of Sun Yat-sen University, Guangzhou 510655, Guangdong, China.; ^2^Guangdong Research Institute of Gastroenterology, The Sixth Affiliated Hospital of Sun Yat-sen University, Guangzhou 510655, Guangdong, China.; ^3^Cancer Precision Medicine, Genome Institute of Singapore, Agency for Science, Technology, and Research, Biopolis, Singapore 138672, Singapore.; ^4^Department of Physiology, Yong Loo Lin School of Medicine, National University of Singapore, Singapore 117597, Singapore.; ^5^Cancer and Stem Cell Biology, DUKE-NUS Graduate Medical School of Singapore, Singapore 169857, Singapore.

**Keywords:** Cancer-associated fibroblasts (CAFs), heterogeneity, therapeutic resistance, CAFs targeting

## Abstract

The “seed and soil” concept has reformed paradigms for cancer treatment in the past decade. Accumulating evidence indicates that the intimate crosstalk between cancer cells and stromal cells plays a tremendous role in tumor progression. Cancer-associated fibroblasts (CAFs), the largest population of stroma cells, influence therapeutic effects through diverse mechanisms. Herein, we summarize the recent advances in the versatile functions of CAFs regarding their heterogeneity, and we mainly discuss the pro-tumorigenic functions of CAFs which promote tumorigenesis and confer therapeutic resistance to tumors. Targeting CAFs is emerging as one of the most appealing strategies in anticancer therapies. The endeavors to target or reprogram the specific subtypes of CAFs provide great cancer treatment opportunities, which may provide a better clinical benefit to cancer patients.

## INTRODUCTION

Most conventional cancer treatment strategies are based on the special characteristics of tumor cells, such as rapid proliferation rate and oncogenic driver mutations in cancer cells. However, few patients experience complete responses, and the strength and duration of response to treatments vary widely. Intrinsic or acquired resistance developing during the treatment is the central issue in cancer therapies, which often leads to tumor progression. Thus, new therapeutic strategies are urgently needed to bypass or overcome drug resistance in cancer treatment. Before that, a profound understanding of the mechanism of resistance is needed.

The “seed and soil” theory, which Stephen Paget proposed first in 1889, has received widespread attention in recent years. Cancer-associated fibroblasts represent the majority of stromal cell populations in the tumor microenvironment (TME) and are closely linked with clinical outcomes across multiple cancers including colorectal cancer (CRC), pancreatic ductal adenocarcinoma (PDAC), and breast cancer^[1-4]^. While numerous studies have revealed that CAFs play pivotal roles in regulating tumor development and progression via various “intermediator messengers” involving extracellular matrix, soluble factors, and metabolites^[5-7]^, bench-to-bedside translation remains the bottleneck for researchers to properly target CAFs as an efficient antitumor therapy. Thus far, no CAF-specific inhibitors have been approved by the United States Food and Drug Administration (FDA), and this might be, at least partially, ascribed to the high heterogeneity of CAFs. 

### Heterogeneity of CAFs origin

CAFs can be derived from various cell populations. Tissue-resident fibroblasts are considered one of the most prevalent precursors for CAFs. Soluble factors such as transforming growth factor-β (TGF-β) and platelet-derived growth factor (PDGF) derived from neighboring tumor cells have been implicated in de novo activation of CAFs^[8,9]^. Additionally, exosomes (including shuttling cargos such as miRNAs and lncRNAs) have also played essential roles in the transformation of normal fibroblasts (NFs) to CAFs. In some types of cancers, such as pancreatic and liver cancers, stellate cells are recognized as another critical source of CAFs, which have been termed pancreatic stellate cells (PSCs) and hepatic stellate cells (HSCs), respectively. Classic TGF-β, PDGF signaling, and vitamin A deficiency have been found to be involved in PSC activation^[10,11]^. Furthermore, a recent work unveiled that stimulation of IGF-1 signaling assisted HSCs in acquiring a fibroblast-like phenotype. Mesenchymal stem cells (MSCs) are also one of the most commonly studied sources of CAFs. Effectors stimulating transdifferentiation of MSCs to CAFs vary across different cancers^[12,13]^. For instance, the OPN-MZF1-TGF-β axis was able to mediate MSC-CAF transformation in breast cancer^[14]^, while TGF-β, as well as CXCL16, participated in the activation of MSCs in prostate cancer. Lastly, other types of cells, including epithelial cells, endothelial cells, hematopoietic stem cells (HSCs), cancer stem cells, adipocytes, and pericytes, have also been reported to possess the potential to transdifferentiate into CAFs. Of note, there is less evidence relating to these origins, which needs further investigation^[15]^. Taken together, the origins of CAFs have not been fully elucidated yet. As distinct effectors/signaling pathways contribute to the generation of CAFs, which are related to cancer types or the cell types that the CAFs originated from, it would be meaningful to monitor the dynamic origins of CAFs more precisely during cancer progression by taking advantage of advanced technologies such as lineage tracing and single-cell special analysis.

### Functional diversity of CAFs

Similar to the existence of heterogeneity in cellular origins, CAFs exhibit diversity regarding their biological characteristics and function, which was firstly corroborated by David A. Tuveson Group^[16]^. By exploiting in vitro 3D co-culture system and in vivo mouse/patient-derived PDAC tissues, they found two distinct subtypes of CAFs present in PDAC. One subpopulation of CAFs, located immediately adjacent to neoplastic cells, showed elevated expression of α-SMA and low expression of IL-6 (myCAFs), whereas the other, distantly distributed throughout the tumor, had reduced α-SMA expression and elevated production of inflammatory factors including IL-6, thus was termed inflammatory CAFs (iCAFs). Intriguingly, these two subpopulations of CAFs showed distinct transcriptome profiles related to their characteristics and could dynamically change from one state to the other. This study highlighted that various subtypes of CAFs rather than one homologous pro-tumoral CAF population may exist in the TME, which can partially explain the failure encountered in clinical trials by targeting α-SMA+ CAFs^[17,18]^. Likewise, the two aforementioned CAF populations with distinct α-SMA expression and transcriptomes have also been reported in CRC, supporting the findings in PDAC^[19]^. Moreover, another study from the same group further identified a novel population of CAFs with MHC-II and CD74 expression termed antigen-presenting CAFs (apCAFs). This new subtype of CAFs can directly activate CD4+ T cells in an antigen-specific fashion, confirming the putative immune-modulatory capacity of CAFs^[20]^. In accordance, more and more in-depth studies have pointed to the functional diversity of these subpopulations of CAFs. For example, pharmaceutical inhibition or genetic ablation of Shh signaling, which is involved in driving myCAF activation in PDAC, resulted in increased metastasis and decreased animal survival^[21,22]^. A similar phenotype was demonstrated in parallel by another group, which also showed depletion of α-SMA+ fibroblasts and led to poorly differentiated tumors and shortened animal survival. More importantly, low myCAF content was found to be associated with worse overall survival in human PDAC tumor sections^[21,23]^. Collectively, this evidence strongly supports the tumor-constraining role of myCAFs, which should always be kept in mind when considering whether to target CAFs as anticancer therapy. On the contrary, accumulating evidence uncovers the pro-tumoral properties of iCAFs. This was not very surprising since the key hallmark of iCAFs is secreting inflammatory factors such as IL-6, which have been well-studied for their tumor-promoting capability^[17]^. Recent work from our lab showed that IL-6 secreted by CAFs can promote LRG1 expression through STAT3-mediated transactivation, which facilitates epithelial-to-mesenchymal transition (EMT) and ultimately leads to liver metastasis in a xenograft mouse model of CRC. Since many agents that target individual nodes of the IL-6/STAT3/LRG-1 cascade, including IL-6, IL-6R, or JAKs/STAT3, are currently under active investigations as treatments for hematopoietic malignancies and solid tumors, this work opens a new and implementable way to mitigate metastasis by blocking CAF-tumor cell crosstalk in CRC^[24]^. Additionally, another seminal study from David A. Tuveson Group revealed the underlying molecular mechanism that promotes the diversity of CAFs. They reported that IL-1 induced LIF expression and downstream JAK/STAT activation to generate iCAFs. Conversely, TGF-β was able to antagonize iCAF generation by downregulating IL-1R expression and promote shifting to myCAFs. Consistently, targeting JAK/STAT signaling reduced the number of iCAFs and increased α-SMA+ myCAFs, indicating a shift from iCAFs to myCAFs. Ultimately, this phenotypic shift within the two subpopulations of CAFs led to a dramatic decrease in tumor volume, confirming the opposite function of the two CAF subtypes. This study raised a promising strategy to tackle cancer by converting pro-tumoral CAFs to tumor-constraining CAFs or selectively depleting tumor-promoting CAFs^[25]^. The functional heterogeneity of cancer-associated fibroblasts in distinct tumors is summarized in detail in Table 1.

**Table 1:** Functional heterogeneity of cancer-associated fibroblasts in distinct tumor types

**Cancer types**	**CAF subtypes**	**Characteristic markers**	**Functions**	**References**
BC	CD10+/GPR77+ CAFs	CD10, GPR77	Chemoresistance, proliferation, migration	[34]
	dCAFs	*SCRG1, SOX9, SOX10*, *etc*.		[86]
	mCAFs	Fibulin-1, PDGFRα		[86]
	vCAFs/cCAFs	Nidogen-2	Angiogenesis	[86]
CRC	CAF-A	*MMP2, DCN, COL1A2, PDPN, FAP*		[88]
	CAF-B	*ACTA2,* *TAGLN, PDGFA, LUM*		[88]
OSCC	CAF-D	TGF-β1	Invasion, EMT	[90]
	CAF-N	Hyaluronan	Invasion	[90]
PDAC	apCAFs^a^	*H2-Aa, H2-Ab1, Cd74, Saa3, Slpi*	Antigen-present, Immunosuppression	[20,58,59]
	iCAFs^a^^,^^b^	*IL6, IL8, PDGFRA, CFD, PLA2G2A, HAS1, CXCL2, CCL2, CLU, EMP1, LMNA*	Immunosuppression, chemoresistance	[17,20,91]
	myCAFs^ a^^,^^b^	*ACTA2, TAGLN, MMP11, MYL9, HOPX, POSTN, TPM1, TPM2*	Proliferation, migration, invasion, ECM remodeling	[17,20,91,92]
	meCAFs	Highly active glycolysis	Higher risk of metastasis and poor prognosis but better response to immunotherapy	[93]
	NetG1+CAFs	Netrin G1	Nutritional support (glutamate/glutamine metabolism), immunosuppression	[74]
PDAC/Oral/CRC/Bladder cancers	rCAFs	Meflin, BMP-4, Hedgehog, IKKβ	Antitumoral effect	[21-23,94-98]

BC: Breast cancer; CRC: colorectal cancer; OSCC: oral squamous cell carcinoma; PDAC: pancreatic ductal adenocarcinoma; EMT: epithelial-to-mesenchymal transition; ECM: extracellular matrix.^ a^This is also found in BC; ^b^this is also found in CRC.

With advances in single-cell sequencing and multi-omics approaches, more and more novel CAF subsets have been unveiled across different cancers, which have been broadly described elsewhere^[18,26]^. Hereafter, to make the content of this review more clinically relevant, we mainly focus on discussing the CAF subpopulations with tumor-promoting properties, thereby possibly being considered as potential targets to overcome therapeutic resistance.

**Figure 1:**
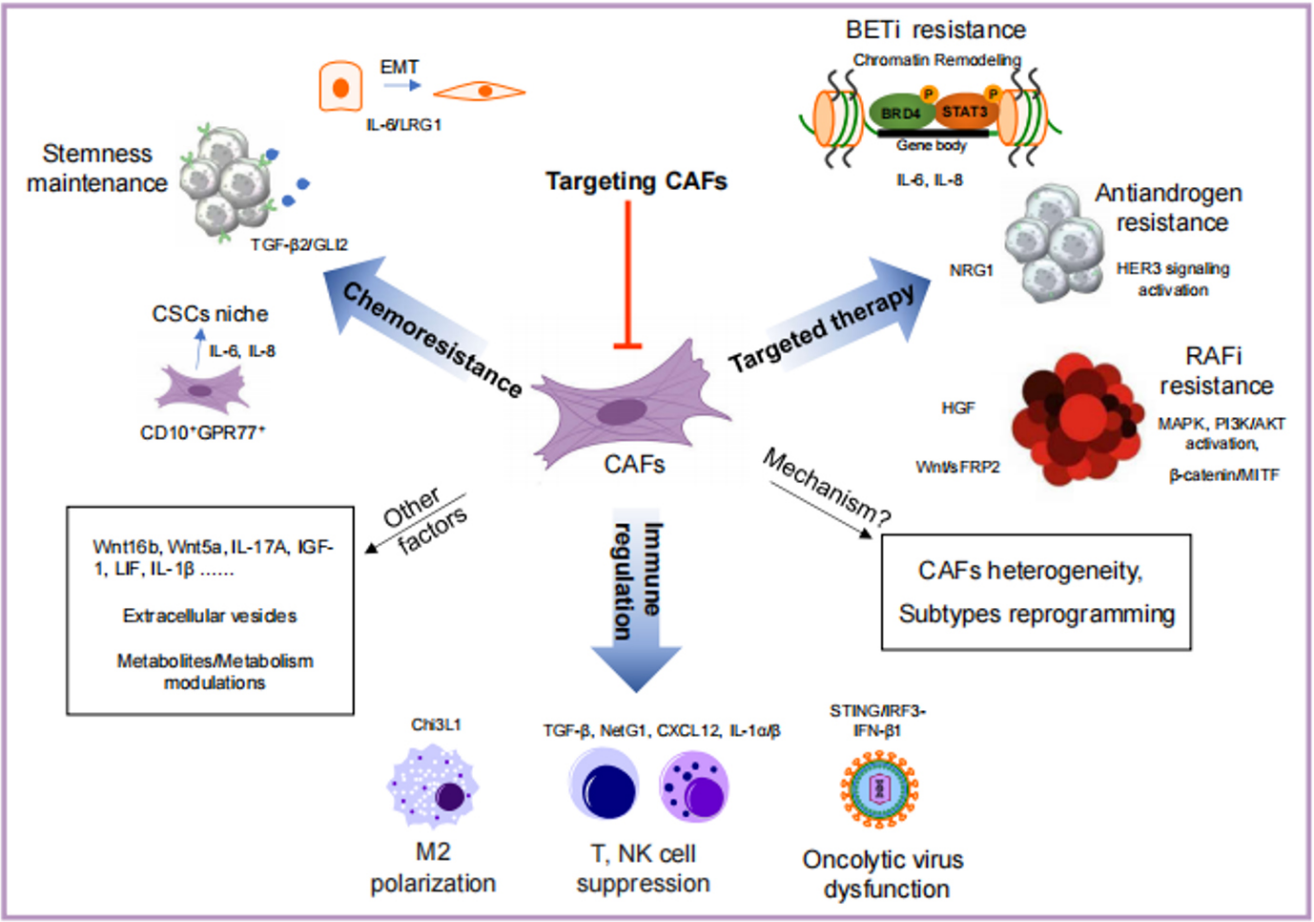
Roles of cancer-associated fibroblasts (CAFs) in cancer drug resistance. CAFs affect almost all cancer treatments, including traditional chemotherapy, targeted therapy, and immunotherapy. There are various mechanisms, such as secretion of growth factors, production of extracellular vesicles, and metabolites, through which CAFs promote drug resistance. Chemoresistance is enhanced by secreted factors from CAFs. Among them, TGF-β2 and IL-6/IL-8 from CD10^+^GPR77^+^ CAFs induce GLI2 upregulation and NF-κB activation, respectively, to maintain the stemness of cancers. IL-6 and/or IL-8 from CAFs were also found to induce EMT or chromatin remodeling in cancer cells. Upon stimulation with cytokines, such as IL-6, IL-8, NRG1, and HGF, the response to targeted therapies can be undermined by BRD4 modification, HER3 signaling activation, and MAPK and PI3K/AKT activation. Furthermore, CAFs mitigate tumor immunity by polarizing macrophages into the M2 phenotype, suppressing the function of NK and T cells and oncolytic viruses through Chi3L1, TGF-β, NetG1, CXCL12, IL-1α/β, and IFN-β1. Although appealing, targeting CAFs remains a big challenge today. The efforts to define CAF subtypes and further decipher their functions in the microenvironment may shed light on discovering new targeting strategies and provide more benefits to cancer patients. EMT: Epithelial-to-mesenchymal transition; CAF: cancer-associated fibroblast.

Conventional cytotoxic chemotherapies, targeted therapies, and the emerging innovative immune checkpoint inhibitors (ICIs) are the mainstays in treating cancer patients. Numerous studies have revealed the essential roles of CAFs in conferring therapeutic resistance through diverse mechanisms [Figure 1], such as remodeling of the extracellular matrix (ECM), maintaining the stemness of cancer stem cells (CSCs), and metabolic reprogramming^[5-7,27]^. More recently, growing evidence also demonstrates the ability of CAFs to modulate tumor immunity^[15,28]^.

### CAFs promote resistance to chemotherapy 

Chemotherapies are still the main first-line treatment strategies for cancer patients. CAFs are one of the most prevalent stromal cell types within the TME across multiple cancers such as CRC and PDAC, and they play pivotal roles in regulating the response to chemotherapy. As the major source of extracellular matrix (ECM) components, CAFs are considered as a physical barrier to infuluence drug delivery. For instance, depletion of CAFs by inhibition of the Hedgehog cellular signaling pathway or administration of hyaluronan was able to enhance the delivery of chemotherapy (gemcitabine) in a PDAC mouse model^[29,30]^. Additionally, by upregulation of lysyl oxidase (LOX) or MMPs, CAFs can alter the abundance and composition of ECM components, especially collagen, ultimately leading to dysregulated ECM homeostasis and resistance to chemotherapy (epirubicin and paclitaxel)^[31,32]^. Cancer stem cells (CSCs) are a small cell population within the bulk tumors that possess self-renewing capability and are largely responsible for resistance to chemotherapy. Through the production of cytokines, chemokines, and exosomes (including shuttling cargos such as miRNAs, lncRNAs, or cirRNAs), CAFs play a pivotal role in regulating cancer stemness and are therefore also a route of therapeutic resistance^[33]^. A recent study revealed that IL-6 and IL-8 were secreted by a unique subset of CAFs expressing both CD10 and GPR77 against multiple chemotherapeutic interventions (doxorubicin, cyclophosphamide, and paclitaxel and docetaxel and cyclophosphamide) in breast and lung cancers. CD10^+^GPR77^+^ CAFs constantly secrete IL-6 and IL-8 through activating the NF-KB signaling pathway, providing a survival niche for cancer stem cells^[34]^. Our recent study also demonstrated that hypoxia-induced HIF1α and CAF-derived TGF-β2/Smad signal concurrently transactivate the expression of Hedgehog transcription factor GLI2 in cancer stem cells. Blockade of GLI2 signal induced by HIF1a and TGFβ2 effectively reverses the CAF-promoted resistance to 5-FU-based chemotherapy in colorectal cancer. Interestingly, a high level of HIF1α/TGFβ2/GLI2 can predict the high risk of recurrence in patients undergoing chemotherapy. This study proposed a potential biomarker as well as a potential new strategy to overcome chemoresistance by targeting CAFs signaling^[35]^. Similarly, by directly transferring exosomes as well as its shuttling cargo-miR-92a-3p to cancer cells, CAFs contributed to cancer stemness by activating the wnt/β-catenin pathway, which ended in resistance to therapy^[36]^. Of note, CAFs displayed a high basal level of autophagy compared to their counterparts in different cancers, such as PDAC, ovarian cancer, and head and neck squamous cell carcinoma (HNSCC), and autophagy could be further induced in response to stimuli from the TME^[37-39]^. It is believed that secretory autophagy promotes cancer survival by providing metabolites or other pro-tumoral effectors, including cytokines and growth factors in the harsh tumor milieu. It was also reported that the elevated level of autophagy in CAFs induced epithelial-to-mesenchymal transition and stemness in tumor cells, thus contributing to metastasis and drug resistance. Furthermore, another important role of stress-induced autophagy in CAFs was recently reported to regulate exosome release^[40,41]^. Thus, the involvement of autophagy in shaping the TME deserves further attention. Targeting autophagy-related core machinery by small molecules might be an effective alternative to deal with chemoresistance caused by CAFs.

### CAFs enhance resistance to targeted therapy 

In addition to chemotherapy, the CAF-derived secretome was also found to mitigate the response to targeted therapies. HGF was found to provide an alternative BRAF-independent mechanism for ERK-MAPK activation to mediate resistance to BRAF-targeted therapies in melanoma^[42]^. Wnt signaling modulated by CAFs has also been implicated in resistance to vemurafenib by attenuating the response of melanoma cells to DNA damage^[43]^. CAF-derived NRG1 confers antiandrogen resistance in prostate cancer by activating HER3 signaling^[44]^. In addition, we recently found that the CAF-derived IL-6/8-JAK2 signaling cascade can promote BRD4 phosphorylation^[45]^, a critical epigenetic regulator in the regulation of cancer cell stemness. BRD4 phosphorylation induces chromatin remodeling, supporting a tumor-promoting transcriptional program and thus leading to BET inhibitor resistance. Given the prominent roles of epigenetic reprogramming in tumorigenesis and tumor progression, the finding paves a new way to more effectively treat CRC by co-targeting epigenetic modulators and CAF-mediated signaling pathways. In the presence of CAFs, tumor cells also displayed resistance to cetuximab, a monoclonal antibody therapy targeting epidermal growth factor receptor (EGFR). A further study ascribed this to increased secretion of EGF from CAFs^[10,46]^. Another recent study demonstrated that ECM remodeling and stiffness features were positively correlated with CAFs activation in CRC patients’ tissue samples. Mechanistically, key components of the renin-angiotensin system (RAS), such as angiotensin II (ANGII) produced by CAFs, are involved in ECM deposition. More importantly, targeting CAF-derived RAS signaling was able to improve response to antiangiogenic therapy (bevacizumab), which was due to reduced ECM stiffness^[47]^. Intriguingly, matrix stiffness can induce autophagy in CAFs by stiffness sensing through the Integrin αV-focal adhesion kinase-AMPKα axis^[48]^, forming a CAF–ECM positive feedback regulatory loop. Collectively, these studies show an intimate connection between CAFs and resistance to targeted therapy. Blocking CAF-related signaling pathways will be a powerful strategy to tackle this tough issue.

### CAFs modulate response to ICI

Immunotherapy, specifically immune checkpoint inhibitors (ICIs), has led to a revolution in cancer treatment paradigms in the past decade. While ICIs have shown effectiveness in multiple cancers such as melanoma and lung cancer, the majority of patients cannot benefit from the treatment, especially those with “cold tumors”, such as PDAC and CRC^[49,50]^. Based on the underlying mechanism of ICI action, several potential markers are proposed to be related to clinical response, including the PD-L1 expression level, specifically on tumor cells and APC cells, immune composition within the TME, neoantigens, tumor mutation burden, *etc*. Recent evidence shows that CAFs are linked to the resistance of ICIs^[15]^. CAFs can modulate the recruitment and activity of immune cells mainly through regulating ECM remodeling, the expression of immune checkpoints, and cytokines/chemokines, thereby skewing the TME to immunosuppressive status. For example, CAF-modified ECM is involved in the exclusion of cytotoxic T cells (CTLs) from the proximity of tumor cells. The secretion of matrix proteins and the production of matrix metalloproteinases (MMPs) by CAFs increased matrix stiffness, which not only promotes the migration and invasion of cancer cells but also serves as the physical barrier for immune cell infiltration^[51,52]^. Depletion of FAP+ CAFs, which exhibited upregulation of proinflammatory factors similar to iCAFs, can decrease tumor volumes in a CD4+ T cell- and CD8+ T cell-dependent manner in a KPC mice model^[53,54]^. Treating the FAP+ CAF-depleted mice with ICIs targeting PD-L1/CTLA-4 dramatically reduced tumor volumes. Furthermore, FAP+ CAFs are considered the principal source of CXCL12 and IL-6, which have been implicated in the prevention of T cell accumulation/activity in the tumor. Combined treatment with inhibitor targeting the CXCL12–CXCR4 axis or IL-6 antibody and anti-PD-L1 elicited synergistic efficiency in a PDAC mouse model^[55-57]^. apCAFs, which present antigens to CD4+ T cells through expressing MHCII molecules, were speculated to deactivate CD4+ T cells by inducing either anergy or differentiation into Tregs and dampen antitumor immunity^[20,58]^. Interestingly, an analog to apCAFs was reported to kill CD8+ T cells in an antigen-dependent manner via PD-L2 and FASL^[59]^. Thus, targeting apCAFs might enhance antitumor immunity by restricting immune checkpoint activation. Another newly published work unraveled that CAF-derived wnt2 suppressed dendritic cell (DC) differentiation as well as DC-mediated antitumor T cell response. Targeting wnt2+ CAFs via monoantibody was able to significantly restore antitumor T cell response and enhance response to anti-PD-1 in both esophageal squamous cell carcinoma (OSCC) and a CRC mouse model^[60]^. Additionally, numerous studies have demonstrated that the expression of immune checkpoints such as PD-L1, PD-L2, and B7-H3 on CAFs can directly induce T cell exhaustion and deactivation^[59,61,62]^. Moreover, CAFs were reported to induce PD-L1, PD-1, cytotoxic lymphocyte-associated antigen-4 (CTLA-4), lymphocyte-activation gene-3 (LAG-3), and mucin-domain containing-3 (TIM-3) on the surface of immune cells or tumor cells, which dampen the proliferation and activity of immune cells, especially cytotoxic T cells^[63-66]^. By regulating the expression of those immune checkpoint molecules, CAFs also possibly potentiate the effect of ICIs. Enhanced recruitment of immunosuppressive cells, such as tumor-associated macrophages (TAMs) and myeloid-derived suppressor cells (MDSCs), was also shown to reduce the sensitivity of immunotherapy^[67-69]^. For instance, overexpression of proline isomerase (PIN1) in CAFs was correlated with more infiltration of TAMs and fewer infiltrated CD8+ T cells in human PDAC tissue samples. Targeting pin1 rendered PDAC tumors more sensitive to anti-PD-1 treatment by disrupting the immunosuppressive TME^[70]^. CAF-derived soluble effectors such as CCL2 and CSF1 were critical for the recruitment of MDSCs. Depletion of MDSCs by targeting CSF1 was shown to significantly improve response to ICIs such as anti-CTLA4^[53,67,71,72]^. By taking advantage of single-cell analysis, a recently published seminal work has revealed a positive feedback loop between specific CAF-S1 clusters and Tregs in breast cancer, which subsequently contributed to resistance to immunotherapy^[73]^. In addition, a recently identified subtype of CAFs, termed NetG1+ CAFs, possessed intrinsic immunosuppressive properties and inhibited NK cell activity in PDAC^[74]^. Cardiotrophin-like cytokine factor 1 (CLCF1) derived from CAFs was able to promote infiltration and polarization of neutrophils in HCC^[75]^. Taken together, these studies suggest that co-targeting CAFs or CAF-derived signaling pathways might be one of the most attractive options to improve the efficiency of ICIs by reshaping the tumor immune microenvironment.

### Challenges and Perspectives to target CAFs

**Figure 2:**
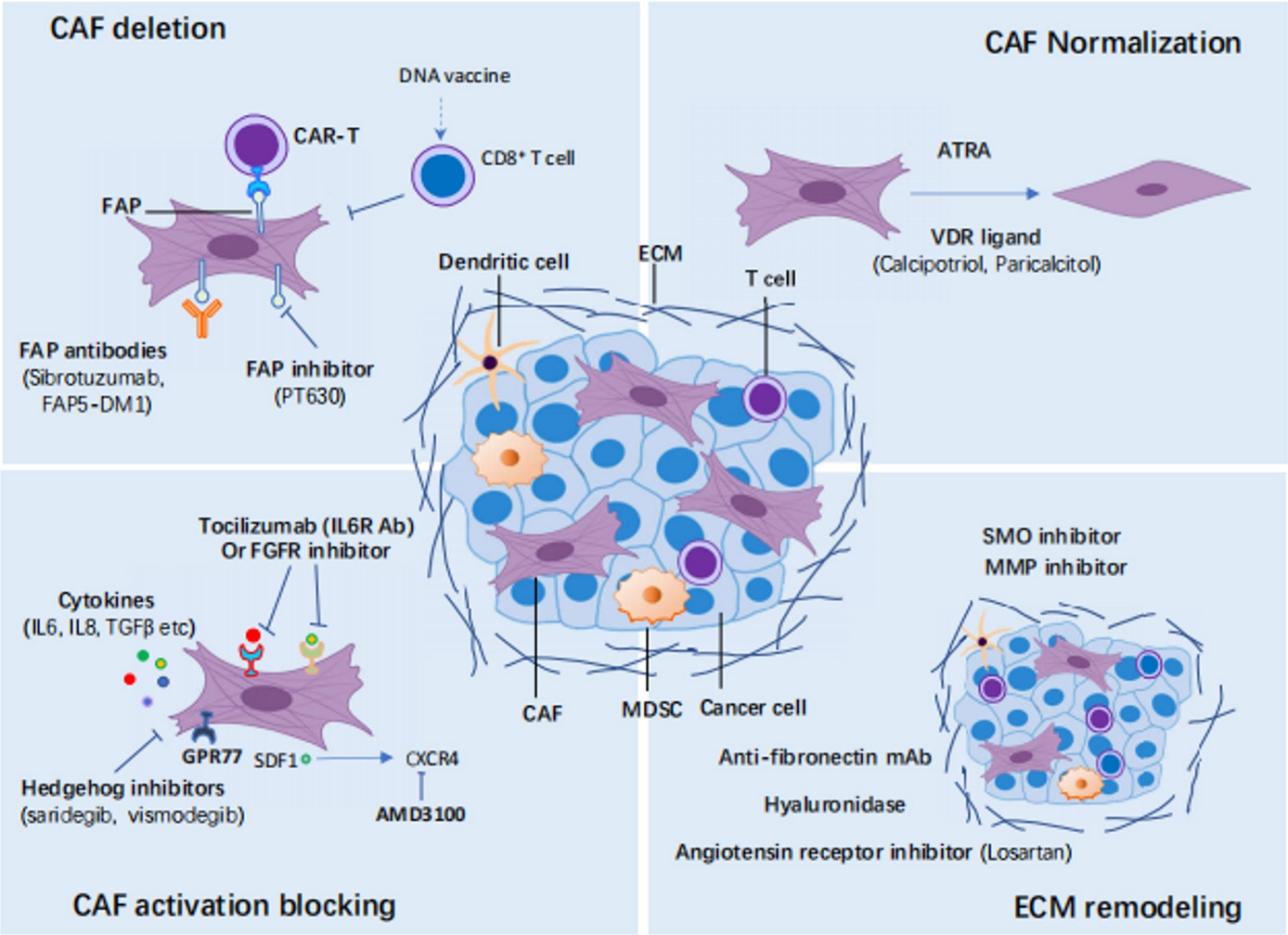
Targeting cancer-associated fibroblasts (CAFs) in cancer. Four main strategies targeting cancer-associated fibroblasts (CAFs) as cancer treatment are discussed. CAFs can be depleted by several treatments targeting CAF-specific markers, such as FAP and α-SMA. The normalization of CAFs from a pro-tumorigenic status to a quiescent or tumor-suppressive state can also be used for cancer treatment with small molecules such as ATRA or VDR ligands. The crucial signalings for CAF tumor-promoting function, such as cytokines and growth factors signalings, can be targeted to inactivate CAFs. Finally, CAF-derived extracellular matrix (ECM) proteins or related signalings can be targeted to induce ECM remodeling. MDSC: Myeloid-derived suppressor cell; FAP: fibroblast activation protein; CAR: chimeric antigen receptor; ATRA: all-trans retinoic acid; VDR: vitamin D receptor; FGFR: fibroblast growth factor receptor; SMO: smoothened; MMP: matrix metalloproteinase.

Although tremendous efforts have been made to either directly or indirectly target CAFs [Figure 2], many strategies have failed to show promising clinical outcomes. The breadth of CAF functions and the interconvertibility of different subtypes pose a challenge for the field. In addition to their oncogenic functions, it has been revealed that CAFs can also play important roles in restraining tumors^[21,23]^. In several clinical or preclinical studies, targeting CAFs by some approaches did not lead to sufficient therapeutic efficacy or even promoted disease progression^[76-78]^. For example, depletion of α-SMA+ CAFs resulted in unexpected immunosuppression and aggressive tumor. Analogously, targeting the Sonic hedgehog (SHH) - smoothened (SMO) signaling that is involved in the activation of myCAFs also did not present therapeutic efficiency or, in some contexts, even shortened patient survival in clinical trials. Further studies indicated that this might be due to the aforementioned heterogeneity of CAFs present in the tumor milieu, and myCAFs tended to play a tumor-restraining role. On the contrary, depletion of FAP+ CAFs or interference with its derived CXCL12-CXCR4 axis restored antitumor immunity in PDAC. This prompted an ongoing phase II clinical trial involving patients with pancreatic cancer (NCT02826486). Thus, more efforts should be addressed to define CAF subtypes and further decipher their functions when interacting with other components in the microenvironment. In addition to direct depletion of CAFs, modulation of CAF activity would be another way to target CAFs. An important study described above found that the IL-1/JAK/STAT signaling cascade was mainly responsible for the generation of iCAF, which displayed tumor-promoting properties across multiple cancers. Targeting IL-1 or JAK was considered as an appealing approach to converting pro-tumoral CAFs into a tumor-restraining subpopulation. The preclinical data have encouraged an early phase I clinical trial to combine standard chemotherapy and IL-1 receptor antagonist Anakinra in PDAC (NCT02021422). All-trans retinoic acid (ATRA) may normalize the CAFs to an inactive state in pancreatic ductal adenocarcinoma (PDAC)^[79]^ (NCT03307148, NCT00001509). Pharmacological stimulation of the vitamin D receptor (VDR) with its ligand calcipotriol can induce stromal reprogramming, ultimately reversing chemotherapeutic resistance induced by CAFs in the PDAC models^[80]^. Targeting the crucial signalings for CAFs’ tumor-promoting function, such as tocilizumab targeting IL-6 receptor^[81]^ (NCT02767557) or an FGFR inhibitor^[82]^ (NCT02699606, NCT01962532, NCT01703481, NCT02421185), can also be exploited to counteract the pro-tumoral effects of CAFs. Finally, CAF-derived extracellular matrix (ECM) proteins or related signalings can be targeted to induce ECM remodeling, which ultimately alleviates therapeutic resistance caused by CAFs. For example, losartan, an angiotensin inhibitor, can reduce hyaluronan production by CAFs, thereby improving vascular perfusion and drug delivery in breast and pancreatic cancers^[83]^ (NCT04106856). The above-mentioned clinical trials targeting CAFs are summarized in Table 2.

**Table 2:** CAF targeting strategies in cancers and related clinical trials

**Target**	**Drugs/agents**	**Cancer models**	**Mechanism of action**	**Phase**	**Trail No.**
CAF depletion					
FAP	FAP antibody (Sibrotuzumab)	Lung cancer	Depletion FAP^+^ CAFs	Phase I	NCT02209727
	Talabostat (PT-100)	Multiple cancer types	Inhibits FAP enzymatic activity	Phase I-II	NCT00303940, NCT00086203, NCT00083252, NCT00083239, NCT00080080
CAF activation blocking					
Hedgehog	IPI-926 (saridegib) and GDC0049 (vismodegib)	Pancreatic Cancer	Reduced CAF activation	Phase I-II	NCT01130142, NCT01195415
	LDE225 (sonidegib)	Multiple cancer types	Inhibits Hedgehog signaling through SMO inhibition	Phase I-II	NCT02027376, NCT02195973, NCT02138929, NCT01487785, NCT01327053, NCT01708174, NCT01350115, NCT00961896
CXCR4	Plerixafor	PDAC, Ovarian and Colorectal Cancer	Inhibit CXCL12 production, restore antitumoral immunity	Phase I	NCT02179970
		Children Cancer, Solid Tumor		Phase II	NCT01225419
	BL-8040	PDAC		Phase II	NCT02826486
IL-6 receptor	Tocilizumab	Pancreatic Carcinoma		Phase II	NCT02767557
FGFR	JNJ-42756493 (erdafitinib)	Lymphoma, Adenocarcinoma, *etc*.	Prevents CAF activation	Phase I	NCT01962532, NCT01703481,
		Multiple cancer types		Phase II	NCT02699606
		Hepatocellular Carcinoma		Phase I-II	NCT02421185
CAF Normalization					
IL-1 receptor	Anakinra	PDAC	CAF normalization	Phase I	NCT02021422
Vitamin A metabolism	ATRA	PDAC, Nephroblastoma	Normalize stellate cells	Phase I-II	NCT03307148, NCT00001509
VDR	Calcipotriol	Breast Cancer,	CAF normalization	Phase I	NCT03596073
	Paricalcitol	Multiple cancer types	CAF normalization and improved chemotherapeutic efficacy	Phase I-II	NCT00637897, NCT03520790, NCT03883919, NCT03415854
ECM remodeling					
Angiotensin receptor	Losartan	Breast cancer, Pancreatic cancers	Reduces hyaluronan production by CAFs	Phase III	NCT04106856, NCT03900793, NCT01805453

PDAC: Pancreatic ductal adenocarcinoma; CAF: cancer-associated fibroblasts.

The rapid development of single-cell RNA sequencing provides great opportunities to define subtypes and interpret the roles of CAFs. Although the high heterogeneity and diverse potential functions of CAFs have been revealed in many types of cancer utilizing scRNA-seq^[20,84-88]^, there are still many details to be elucidated: (1) The precise roles of various CAF subtypes in therapeutic resistance remain largely undefined. The precise identification and characterization of CAFs’ role in promoting or restraining tumors might pave new ways to target CAFs; (2) the mechanism of how the homeostasis between fibroblasts [including CAF subtypes and normal fibroblasts (NFs)] is maintained requires further investigation. For example, fibroblasts play different roles in tumors by secreting different cytokines^[35]^. The interconversion between CAF subtypes might change the tumor’s behavior. Furthermore, NFs can be educated to be tumor-promoting by tumor cells or CAFs^[5,89]^. Epigenetic and chromatin remodeling could be a potential mechanism to interpret these conversions. (3) The effect of therapeutic approaches on CAFs is another direction to be investigated. Many therapeutic paradigms such as chemotherapy could profoundly affect the CAFs’ status and the tumor microenvironment in which they reside, ultimately changing the response of the tumors to treatments. With advances in technologies, the studies of multi-omics such as transcriptomics, proteomics, epigenomics, and metabonomics, improve our understanding of cancer biology in an unprecedented way. A comprehensive analysis and precise functional studies in CAFs are required to integrate these multi-omics data using multiple model systems, especially at single-cell resolution. Next, super-resolved spatial omics studies may offer systematic approaches to understand the interplay between CAFs and other cells in tumors. In conclusion, these comprehensive investigations may warrant both preclinical and clinical studies targeting CAFs to achieve better clinical benefits for patients.

## DECLARATIONS

### Copyright

© The Author(s) 2022.
